# The effects of at-home whitening on patients’ oral health, psychology, and aesthetic perception

**DOI:** 10.1186/s12903-018-0668-2

**Published:** 2018-12-11

**Authors:** Cristian Bersezio, Javier Martín, Andrea Herrera, Alessandro Loguercio, Eduardo Fernández

**Affiliations:** 1 Department of Restorative Dentistry, Faculty of Dentistry - University of Chile, Sergio Livingstone Pohlhammer 943, Independencia -, Santiago, Chile; 2 Universidad Estadual de Ponta Grossa – UEGP, Rua Carlos Cavalcanti, 4748, Bloco M, Sala 64A, Uvaranas, Ponta Grossa, Paraná 84030-900 Brazil; 3 Instituto de Ciencias Biomédicas -Universidad Autónoma de Chile, Pedro de Valdivia 425, Providencia Santiago, Chile; 4 Basic Sciences Institute - Faculty of Dentistry – University of Chile, Sergio Livingstone Pohlhammer 943, Independencia -, Santiago, Chile

**Keywords:** Whitening teeth, Quality of life, OHIP, PIDAQ, OES

## Abstract

**Background:**

The aesthetic self-perception and psychosocial impact of whitening is poorly explored in the literature; it is even less clear whether the effect of whitening may influence the psychology or social relations of patients. Therefore, the aim of this trial is to evaluate the impact of an at-home whitening procedure on patients’ quality of life. Also, this study’s secondary objective is the adaptation and validation of the Spanish version of the OES questionnaire (OES-SP).

**Methods:**

Fifty eight patients underwent whitening with 10% carbamide peroxide (CP) gel for 1 h daily for 3 weeks. For these participants, the OHIP-Esthetics, PIDAQ, and OES surveys were applied before, one week after, and one month after the whitening procedure. Oral health-related quality of life was measured using the OHIP-Esthetics survey and the psychosocial impact using the PIDAQ survey. The orofacial aesthetics was measured by OES and validated for the Spanish Chilean context. The treatment efficacy (ΔE and ΔSGU) and data from the OHIP-Esthetics PIDAQ and OES surveys were compared using the Wilcoxon Signed-Rank test.

**Results:**

The whitening procedure was effective (ΔE = 5.9 ± 1.8). The OHIP-Esthetics results were significant when comparing the initial baseline survey prior to the treatment and one week after whitening (*p* < 0.001) and when comparing the baseline and one-month results (p < 0.001). The overall score on the PIDAQ, after one week post-whitening, was separated into factors and all factors were statistically significant (*p* < 0.03); the factors for the one-month results were also all statistically significant (p < 0.001), except the social impact factor. The OES questionnaire had statistically significantly increased scores both one week and one month post-treatment (*p* < 0.0001). The reliability, validity, and responsiveness of the OES-SP were satisfactory.

**Conclusion:**

The at-home whitening procedure with carbamide peroxide 10% had a positive effect on patients’ oral health-related quality of life, psychology, and aesthetic perception after one month post-whitening. The Chilean Spanish version of the OES showed satisfactory psychometric characteristics to evaluate dental whitening.

**Trial registration:**

NCT02605239. Date that the study was retrospectively registered: 11-11-2015.

## Background

The FDI’s new definition of oral health is related to the physical, mental, and psychosocial well-being of every person, which explains why dental treatments are relevant for people’s quality of life and their psychological and emotional well-being [1]. The emergence of cosmetic dentistry, which seeks to solve problems that affect patients’ self-esteem and their quality of life, fits in directly with the WHO’s definition oh health.

The effect of these aesthetic treatments on a patient’s quality of life, −psychosocial well-being, and aesthetic perception is unclear, and the field lacks an evaluation or weighting of “aesthetics sufficiency” that considers and includes the views of the patients. There are a few reports that attempt to explain this result, but it is not yet fully clear [2–4]. For example, several clinical reports show the effectiveness of dental whitening and its direct relationship with a 5-unit variation of delta E [5], which was either instituted arbitrarily or due to the concept that human beings cannot perceive variations of delta E smaller than 3 units [6]. However, from an individual point of view, bleached patients are best-suited to state whether or not the whitening procedure is considered effective (related to self-perception). Therefore, this definition of effectiveness is incomplete, unclear, or almost entirely related to numerical standards.

Regarding quality of life, dental implants, orthodontic treatment, and periodontal therapy have been associated with improved quality of life in dentistry [7], but our results suggested that dental whitening did not affect participants’ quality of life. Studies related to how whitening influences quality of life are controversial, with some studies showing that dental whitening improves patients’ quality of life [8] and others that agree with the present study, i.e., showing that dental whitening has no impact on patients’ quality of life [9, 10]. This difference can be attributed to the different instruments used to evaluate quality of life (specific and general instruments).

The psychosocial impact of whitening is poorly explored in the literature, and it is even less clear whether whitening may influence the psychology or social relations of patients; the literature indicates that other aesthetic treatments, such as surgery and orthodontics [2, 11], have an effect on these factors. Recently, Fernandez et al. [12] reported a positive impact of whitening on psychological and psychosocial factors, as measured by PIDAQ, 9 months after the whitening procedure. Pleasant teeth play an essential role in social interactions; they can influence achievements and success in relationships, self-confidence, the availability of opportunities, personality evaluations, and possible employment. Aesthetics are not absolute, but extremely personal and subjective, though they are also influenced by their social environment. Facial attractiveness correlates with self-esteem and is equally important for men and women; some studies that describe an improvement in confidence with procedures related to aesthetic improvements in patients. The OES, a scale that was originally developed to assess aspects of orofacial aesthetics, represents a onedimensional instrument that consists of eight items, self-reported by a 5-point Likert-type scale. The OES was developed by Pernilla et al. [13] and has been translated and validated in some languages [14–17], but not Spanish. One goal of this work is to adapt, validate, and apply the Spanish adaptation of the OES questionnaire in this cohort (OES-SP).

Therefore, the primary aim of this study is to evaluate the impact of an at-home whitening procedure on patients’ quality of life, as measured by the OHIP-14, and psychosocial well-being, as measured by the PIDAQ. The study’s secondary aim is to apply, after adaptation and validation, the Spanish version of the OES questionnaire (OES-SP) in the same population to evaluate the patients’ aesthetic self-perception after whitening. The null hypothesis of this paper is that the at-home whitening procedure has no impact on the patients’ quality of life, psychosocial well-being, or aesthetic self-perception.

## Methods

This clinical trial was approved by the Ethical Scientific Committee of the Faculty of Dentistry, Universidad de Chile (trialnumber 13/18a) and registered (NCT02605239). All patients were told about benefits and/or possible adverse effects of the treatment and read and accepted an informed consent form.

Fifty-eight voluntary patients, 18 to 76 years old, were included in the study. All of them had good oral health and at least one central incisor colored A2 or darker, compared to a value-oriented Vita Classical shade guide (Vita classical; Vita Zahnfabrik, Bad Säckingen, Germany). We excluded from the study patients who: had received previous whitening treatment; were undergoing orthodontic treatment; were pregnant or breastfeeding; had bruxism; had non-carious cervical lesions in their anterior teeth, full crowns, restorations over the labial surface or with gingival recession; suffered from spontaneous tooth pain; or had endodontically-treated teeth or internal discolorations.

The study’s sample size was calculated based on previous studies [16–18]. A difference of 3 units in the overall scores of the Esthetics Oral Health Impact Profile questionnaire (OHIP-Esthetics), the Psychosocial Impact of Dental Aesthetics Questionnaire (PIDAQ), or the Oral Esthetic Scale (OES) were considered to be clinically important. Considering a significance level of 5% and a power of 90%, a minimum sample of 58 participants was determined.

All patients were told about benefits and/or possible adverse effects of the treatment and read and accepted an informed consent form. Before starting the whitening treatment, all participants were given 30 min to answer the OHIP-Esthetics [19], PIDAQ [11], and OES [13] questionnaires.

OHIP-Esthetics questionnaire: Oral health-related quality of life was measured using the Chilean Spanish-validated OHIP-Esthetics questionnaire (Table 1) [20]. It is a 14-statement form, where each announcement is accompanied by a 5-point Likert-type scale (where 0 = never, 1 = hardly never, 2 = occasionally, 3 = fairly often, and 4 = very often). Individual scores were added to get the OHIP-Esthetics score, which ranged from 0 to 56. Outcomes were considered the sum of the OHIP-Esthetics score and the result of each of the 7 evaluated dimensions.

**Table 1:** OHIP-Esthetics, PIDAQ and OES questionnaires

OHIP-Esthetics Questionnaire	
Q1 Have you noticed a tooth which doesn’t look right?^1^	
Q2 Have you felt that your appearance has been affected by problems with your teeth?^1^	
Q3 Have you had sensitive teeth for example to heat or to cold food or drinks?^2^	
Q4 Have you had painful areas in your mouth?^2^	
Q5 Have you been self-conscious because of your teeth?^3^	
Q6 Have you felt uncomfortable about the appearance of your teeth?^3^	
Q7 Have you felt that your food is less tasty because of problems with your teeth?^4^	
Q8 Have you avoided smiling because of problems with your teeth?^4^	
Q9 Have you found it difficult to relax because of problems with your teeth?^5^	
Q10 Have you been a bit embarrassed because of problems with your teeth?^5^	
Q11 Have you been less tolerant of your spouse or family because of problems with your teeth?^6^	
Q12 Have you had difficulties doing your usual job because of problems with your teeth?^6^	
Q13 Have you been unable to enjoy the company of other people very much because of problems with your teeth?^7^	
Q14 Have you felt that life in general was less satisfying because of problems with your teeth?^7^	
PIDAQ Questionnaire	
Dental Self-Confidence	
1. I am proud of my teeth.	
2. I like to show my teeth when I smile.	
3. I am pleased when I see my teeth in the mirror.	
4. My teeth are attractive to others.	
5. I am satisfied with the appearance of my teeth.	
6. I find my tooth position to be very nice.	
Social Impact	
7. I hold myself back when I smile so my teeth don’t show so much.	
8. If I don’t know people well I am sometimes concerned what they might think about my teeth.	
9. I’m afraid other people could make offensive remarks about my teeth.	
10. I am somewhat inhibited in social contacts because of my teeth.	
11. I sometimes catch myself holding my hand in front of my mouth to hide my teeth.	
12. Sometimes I think people are staring at my teeth.	
13. Remarks about my teeth irritate me even when they are meant jokingly.	
14. I sometimes worry about what members of the opposite sex think about my teeth.	
Psychological Impact	
15. I envy the nice teeth of other people.	
16. I am somewhat distressed when I see other people’s teeth.	
17. Sometimes I am somewhat unhappy about the appearance of my teeth.	
18. I think most people I know have nicer teeth than I do.	
19. I feel bad when I think about what my teeth look like.	
20. I wish my teeth looked better.	
Aesthetic Concern	
21. I don’t like to see my teeth in the mirror.	
22. I don’t like to see my teeth in photographs.	
23. I don’t like to see my teeth when I look at a video of myself.	
Orofacial Esthetic Scale OES	
How do you feel about the appearance of your face, your mouth, your teeth and your replacements (prostheses, crowns, bridges and implants)?	
0 is ‘Very dissatisfied’ and 10 is ‘Very satisfied’	
1. Your facial appearance.	
Apariencia de su cara	
2. Appearance of your facial profile	
Apariencia de su perfil	
3. Your mouth’s appearance (smile, lips and visible teeth)	
Aspecto de su boca (sonrisa, labios y dientes visibles)	
4. Appearance of your rows of teeth	
Aspecto de la arcada de sus dientes	
5. Shape/form of your teeth	
Forma de sus dientes	
6. Colour of your teeth	
Color de sus dientes	
7. Your gum’s appearance	
Aspecto de sus encías	
8. Overall, how do you feel about the appearance of your face, your mouth, and your teeth	
En general, ¿Cómo se siente sobre la apariencia de su rostro, su boca y dientes?	
1–7: summary score 8: overall impression score	

On OHIP-Esthetics questionnaire numbers correspond to the dimensions (1 = Functional limitation, 2 = Physical pain, 3 = Psychological discomfort, 4 = Physical disability, 5 = Psychological disability, 6 = Social disability, 7 = Handicap). In italic translation to Spanish

PIDAQ is a 23-item questionnaire [21] (Table 1) that measures 4 different components: dental self-confidence, social impact, psychosocial impact, and aesthetic concern [11]. Items must be answered using a 5-point Likert scale (where 0= “not at all”, 1 = “a little”, 2 = “somewhat”, 3 = “strongly” and 4 = “very strongly”). Outcomes were considered the sum of the PIDAQ score and the result of each of the 4 components. Internal consistency was evaluated using Cronbach’s alpha test.

The Orofacial Esthetics Scale (OES) is a scale that was originally developed in the Swedish language [13] and was later translated into English (OES-E) [16] (Table 1). It is an eight-question scale that asks questions regarding the appearance of one’s face, profile, mouth, tooth, alignment, tooth shape, tooth color, and gums, as well as an overall impression of one’s orofacial aesthetics. Each question is accompanied by an 11-point scale, where 0 means “very dissatisfied” and 10 “very satisfied.” The scores of the first 7 items are added to a summary score, and thus the results can range from 0 (very dissatisfied with aesthetics) to 70 (patient very satisfied with all oral aesthetics items). The eighth item (overall impression of orofacial aesthetics) is judged separately and ranges from 0 to 10. Lower scores indicate more impaired orofacial aesthetics.

Initially, the OES was translated into Spanish. For this, a translator with good skills in dental vocabulary in Spanish translated the 8 items of the OES-E; additionally, three dentists who were native Spanish speakers with good English language skills collaborated in the translation of some expressions. Finally, the translated version was reviewed and edited by two other academics with excellent knowledge of the English language (Dental School, Faculty of Dentistry, University of Chile). This editing was done individually and later integrated to get a definite draft that was back-translated into English by a qualified translator in collaboration with the same three previously-consulted native Spanish speakers. The final version of the Spanish OES (OES-SP) was translated back to English and then reviewed by a native English speaker and by two academics from the Faculty of Dentistry with expertise in the English language. After all of them confirmed that there were no significant differences between OES-SP and OES-E, the translation was considered satisfactory and ready for use.

OES-SP cross-cultural adaptation: Before applying the scale to the project’s participants, it was tested in 30 patients (ages: 27 to 63 years old) to ensure its usefulness. Individuals were asked to inform the researchers of any difficulty in understanding or answering the questionnaire. To avoid misunderstanding about what the scale assessed, items like “your facial appearance” and “appearance of your facial profile” were modified to “appearance of the lower-third of your face” and “appearance of the lower-third or your facial profile” as the scale is designed to evaluate the aesthetics of the lower-third of the face and teeth.

Statistical analysis to validate OES-SP: reliability (internal consistency and test-retest), validity (construct, convergent, discriminative), and responsiveness were assessed.

Internal consistency was determined using the average inter-item correlation and Cronbach’s alpha coefficient at the three application moments (before treatment and one week and one month after treatment). The average inter-item correlation was considered to be more than 0.40. Cronbach’s alpha values over 0.75 correspond to excellent outcomes; values between 0.75 and 0.40 are considered satisfactory and values below 0.40 correspond to poor outcomes.

Test-retest reliability was determined as temporal stability by the interclass correlation coefficient (ICC) using a one-way analysis of variance. Values of ICC lower than 0.40 indicated poor correlation, values between 0.41 and 0.60 moderate correlation, between 0.61 to 0.80 good correlation, and over 0.81 excellent inter-class correlation.

Construct, convergent, and discriminative validity were tested in all participants. The exploratory factor analysis (EFA) was used to assess the number of factors in the OES-SP. The Kaiser-Mayer-Olkin (KMO) test, Bartlett’s test of sphericity, and the scree plot were used. Eight values over 1 was set as the criteria for a factor; also, significant factors loading > 0.30 were defined.

Convergent validity was tested by Spearman’s rank correlation between the self-reported general satisfaction with orofacial aesthetics and the OES-SP summary score.

Discriminative validity assumes that unrelated measures are really not related. It was predicted that non-orofacial anomalies would have better aesthetics than patients with prosthodontic treatment needs.

Responsiveness was measured by asking participants to complete the questionnaire 3 times: before treatment, one week after, and one month after bleaching. Differences between the before-treatment OES-SP summary scores and the one-month after bleaching scores were analyzed using the paired t-test and by calculating the effect size and standardized response mean.

Changes of OES-SP: temporal stability was assessed through the test-retest reliability. The ICC between the one-week control scores and the one-month scores was used to measure reliability.

All three questionnaires were administered by a research operator before bleaching treatment (baseline) and one week and one month after completing the treatment.

Bleaching procedure: after administering the baseline questionnaires, tooth color was registered at the baseline subjectively and objectively. For subjective color evaluation, the Vita Classical shade guide (Vita Zahnfabrik, Bad Säckingen, Germany) was value-ordered from the lightest (B1) to darkest (C4) tab. Although this scale is not linear in the truest sense, it was treated as continuous with a linear ranking [5]. Two calibrated operators (weighted Kappa =0.85) recorded tooth color at the middle area of the labial surface of the upper central incisor, as established by the American Dental Association [22].

Objective color registry was performed using the Vita Easyshade spectrophotometer (Vita Easyshade, Vita Zahnfabrik) [23]. To assure the reading of the same area before and after treatment, a heavy silicone (Coltoflax and Perfil Cub, Vigodent, Rio de Janeiro, Brazil) impression of the maxillary arch was taken, where a 6 mm radius window was created over the middle area of the labial surface of central incisors, where the spectrophotometer tip was inserted. L*, a*, and b* color coordinates were recorded.

Whitening treatment was performed using an at-home 10% carbamide peroxide (CP) gel (Whiteness Perfect, FGM), for at-home whitening it has been reported that the 10% concentration has a similar efficacy with lower risk and intensity of hypersensitivity than higher concentration [24]. Each participant was taught to use the gel for 1 h daily during 3 weeks [25] and to apply it in a whitening tray that was trimmed 1 mm beyond the marginal gingiva. After each use, the tray was removed and cleaned and teeth were brushed as usual with a fluoridated toothpaste with no whitening compounds.

The tooth color registry was repeated during the whitening treatment (after the first, second, and third weeks of treatment) and one week and one month after the end of it. Subjective color changes were calculated as the number of shade guide units change (ΔSGU) over the value-oriented shade guide between each checkpoint and the baseline registry. For objective color change, the difference between each recall period and baseline (∆E) was calculated using the formula (CIE, 1978) ∆E = [(∆L*)^2^ + (∆a*)^2^ + (∆b*)^2^]1/2.

Tooth sensitivity evaluation: during treatment, patients were told to keep a daily registry of tooth sensitivity (TS) using a visual analog scale (VAS) [5, 26, 27]. This was a 100-mm line with the descriptors “no tooth sensitivity” at the left end and “unbearable tooth sensitivity” at the right end. Patients marked the line that they felt corresponded to the intensity of tooth sensitivity and the distance from the left end to the mark was measured.

Data collection and statistical analysis: treatment efficacy data (ΔE and ΔSGU) were tested for normality and homogeneity of the variance-covariance matrix by the Kolmogorov-Smirnov test. As data were not normally distributed, changes in color between baseline and post-treatment evaluations were analyzed using the Wilcoxon Signed-Rank Test.

The absolute risk of tooth sensitivity was considered as the percentage of patients who experienced sensitivity at least one time during the bleaching therapy. It was reported with a 95% confidence interval. The intensity of tooth sensitivity (by the VAS scale) data are reported as the mean and standard deviation.

Data from the OHIP-Esthetics, PIDAQ, and OES questionnaires were separately collected in a spreadsheet and analyzed by a blind operator. All statistical analyses were performed on SPSS 24.0 (SPSS Inc., Chicago, IL, USA) considering a level of significance of 5% (α = 0.05).

## Results

The demographic characteristics of the participants from both groups are presented in Table 2. The results of effectiveness by ΔSGU and ΔE in the different times and their comparisons are in Table 3.

**Table 2:** Baseline features of the participants. SD = standard deviation

Baseline features	
Age (years; means ± SD)	28.8 ± 9.1
Minimum age (years)	19
Maximum age (years)	55
Male (%)	50
Baseline SGU (mean ± SD)	7.26 ± 1.7
L (mean ± SD)	84.4 ± 3.84
a (mean ± SD)	−0.12 ± 0.63
b (mean ± SD)	22.00 ± 2.8

**Table 3:** Color change in ΔSGU and ΔE in the different assessment points

Assessment points	Color change	
	ΔSGU	ΔE
Baseline vs. 3-week bleaching	2.7 ± 1.5 a	6.4 ± 3.4 A
Baseline vs. 1-week after bleaching	2.6 ± 1.6 a	5.9 ± 1.9 A
Baseline vs. 1-month after bleaching	2.4 ± 1.7 a	5.9 ± 1.8 A

Comparisons are valid only within columns. The same letter indicates statistically similar means *(p > 0.05*

### Whitening-induced tooth sensitivity

Thirty out of the 58 participants submitted to whitening reported pain at least once during whitening. Therefore, the absolute risk of sensitivity was 51.7% (95% confidence interval of 39.2 to 64.1%). In general, the tooth sensitivity was mild, with a mean intensity of 0.8 ± 1.2 by the VAS scale.

### OHIP-esthetics

The OHIP-Esthetics survey scores were significant when comparing the initial baseline survey prior to the treatment to the one-week (*p* < 0.001) and one-month (p < 0.001) after whitening scores. Specifically, we had statistically significant differences after one week in the following dimensions: functional limitation, psychosocial discomfort, physical disabilities, psychological disabilities, and handicap as well as in the overall score. After one month post-whitening, we observed a statistically significant difference in all factors as well as in the overall score (*p* < 0.04) (Table 4).

**Table 4:** Distribution of scores by dimension and for the total OHIP-14 (Oral Health Impact Profile in Spanish) expressed in mean and SD

	Baseline	1-week post bleaching	1-month post bleaching	Corrected item total correlation of sum	Cronbach Alpha’s if Item Deleted
Functional Limitation	5.6 ± 2.3	4.7 ± 1.7*	4.3 ± 1.5*	0.637	0.808
Physical Pain	4.5 ± 1.5	4.2 ± 1.2	4.1 ± 1.3*	0.361	0.842
Psychological Discomfort	5.7 ± 1.7	5.3 ± 1.4*	5.2 ± 1.5*	0.427	0.836
Physical Disability	3.2 ± 1.3	2.8 ± 1.1*	2.7 ± 1.0*	0.732	0.792
Psychological Disability	3.7 ± 1.8	3.3 ± 1.5*	2.8 ± 1.1*	0.792	0.772
Social Disability	2.8 ± 1.4	2.6 ± 1.0	2.4 ± 0.7*	0.604	0.810
Handicap	2.9 ± 1.4	2.5 ± 0.98*	2.4 ± 0.8*	0.677	0.801
**Overal OHIP score**	28.4 ± 8.0	25.4 ± 6.2*	23.9 ± 5.6*		

. * = *p* < 0.05 compared to baseline by Wilcoxon test. Cronbach’s Alpha: 0.833

### PIDAQ

The overall score on the PIDAQ was not statistically significant at any time. At one week post-whitening, the separated factors were statistically significant (*p* < 0.03) and after 1 month, all the factors were statistically significant (*p* < 0.001) except the social impact factor (Table 5).

**Table 5:** Distribution of scores by dimension and for the total PIDAQ expressed in mean and SD

	Baseline	1-week post bleaching	1-month post bleaching	Corrected item total correlation of sum	Cronbach Alpha’s if Item Deleted
Self Confidence	18.7 ± 5.5	21.3 ± 6.1*	22.1 ± 5.4*	−0.727	0.832
Social Impact	16.8 ± 7.5	15.5 ± 7.1*	15.7 ± 8.7	0.571	−2.543
Psychological Impact	16.0 ± 5.2	14.7 ± 5.6*	14.1 ± 5.6*	0.513	−1.088
Aesthetic Concern	7.2 ± 4.1	5.8 ± 3.1*	6.0 ± 3.1*	0.546	−0.627
PIDAQ	58.7 ± 11.1	57.2 ± 11.3	57.9 ± 12.7		

* = *p* < 0.05 compared to baseline by Wilcoxon test. Cronbach’s Alpha: 0.757

### OES-SP

The OES questionnaire score had a statistically significantly increased score at the one week and one month post-treatment periods (*p* < 0.0001). There was an statistically significant difference in the mouth and teeth factors (p < 0.001), but the face factor was not statistically significantly different (> 0.50). In the comparison between the one week and one month post-whitening scores, only the difference in teeth factor scores was statistically significant (< 0.046) (Table 6).

**Table 6:** Distribution of scores by dimension and for the total OES expressed in mean and SD

	Baseline	1-week post bleaching	1 month post bleaching	Corrected item total correlation of sum	Cronbach Alpha’s if Item Deleted
Mouth	26.6 ± 7.1	28.6 ± 6.6*	29.6 ± 5.9*	0.870	0.460
Face	14.7 ± 3.4	14.8 ± 3.3	15.1 ± 3.0	0.452	0.853
Teeth	11.7 ± 3.7	13.6 ± 3.9*	14.4 ± 3.2*	0.768	0.578
OES	53.0 ± 11.9	57.0 ± 11.9*	59.1 ± 10.9*		

Cronbach’s Alpha: 0.772

. * = *p* ≤ 0.05 compared to baseline by Wilcoxon test

#### Reliability, validity, and responsiveness to validate the OES-SP version

##### Reliability

The corrected item-total correlation ranged from 0.363 to 0.831. The lowest coefficient was found for the second question, “Appearance of your facial profile,” and the highest coefficient for the eighth item, “Overall, how do you feel about the appearance of your face,your mouth, and your teeth?” If items were deleted one by one, the Cronbach’s alpha would not increase and it ranged between 0.849 and 0.950 (Table 6). The internal consistency of the OES-SP showed excellent results based on the average intra-class correlation of 0.721 and Cronbach’s alpha values of 0.904 (week recall) and 0.918 (month recall). The test-retest reliability was performed in the DS group (Tables 6 and 7). The ICCs were appropriate and there were no significant differences for the OES total summary score (*p* = 0.127) between the one-week and one-month recall comparisons (Table /).

**Table 7:** Internal Consistency of OES Sp by questions (Q).Cronbach’s Alpha in different times (Baseline = 0.883; one week = 0.944 and;one month = 0.918)

Items	Media of the scale if the item is deleted	Variance of the scale if the item is deleted	Correlation item-total corrected	Cronbach’s Alpha if the item is deleted
BASELINE				
8	45.52	120.605	0.524	0.880
Q2	45.86	124.717	0.363	0.895
Q3	46.38	102.485	0.827	0.849
Q4	46.71	102.667	0.813	0.851
Q5	46.36	109.814	0.729	0.861
Q6	47.93	112.592	0.581	0.876
Q7	46.31	112.358	0.582	0.876
Q8	46.05	111.138	0.831	0.854
ONE WEEK RECALL				
Q1	49.47	118.499	.593	.900
Q2	49.74	123.353	.395	.916
Q3	50.02	103.561	.838	.878
Q4	50.24	98.958	.867	.875
Q5	50.16	102.590	.814	.880
Q6	50.29	108.527	.707	.891
Q7	49.52	118.675	.565	.903
Q8	49.69	112.393	.805	.885
ONE MONTH RECALL				
Q1	52.09	84.483	.708	.910
Q2	52.46	84.071	.511	.929
Q3	52.43	76.940	.888	.894
Q4	52.63	78.675	.843	.898
Q5	52.59	79.628	.750	.906
Q6	52.52	84.945	.610	.917
Q7	52.34	81.283	.737	.907
Q8	52.20	81.761	.867	.899

##### Responsiveness

The questionnaire was administered three times; the first time prior to the treatment, the second time a week after the treatment, and the third time a month after the treatment. As predicted, the OES score significantly increased after the treatment (Table 8).

**Table 8:** Responsiveness OES Sp. The scores of each question and the total score were compared between baseline and post treatment (*)

	Baseline	One week recall		
	Media (SD)	Media (SD)	*p* value	Effect Size
Q 1	7.50 ± 1.76	7.55 ± 1.67	0.661	0.028
Q 2	7.16 ± 1.92	7.28 ± 1.83	0.327	0.063
Q 3	6.64 ± 2.19	7.00 ± 2.05	0.040*	0.164
Q 4	6.31 ± 2.21	6.78 ± 2.25	0.013*	0.213
Q 5	6.66 ± 1.98	6.86 ± 2.16	0.133	0.101
Q 6	5.09 ± 2.16	6.72 ± 2.04	0.000*	0.755
Q 7	6.71 ± 2.18	7.50 ± 1.72	0.001*	0.362
Q 8	6.97 ± 1.71	7.33 ± 1.63	0.022*	0.211
**TOTAL**	**53.02 ± 11.99**	**57.02 ± 11.95**	0.000*	0.334
Face	14.66 ± 3.44	14.83 ± 3.27	0.551	0.049
Mouth	26.62 ± 7.07	28.60 ± 6.56	0.001*	0.280
Teeth	11.74 ± 3.65	13.59 ± 3.87	0.000*	0.507

(*) Wilcoxon test (*p* < 0.05)

The scores of each question and the total score were compared between baseline and post treatment (*)

##### Validity

Factor loadings for each item ranged between 0.748 and 0.941. The Bartlett’s test of sphericity value was 1646.154 (df = 28, *P* < 0.001) and the Kaiser – Meyer – Olkin (KMO) test value was 0.921, more than the critical value of 0.60. Exploratory factor analyses exposed the one-factor structure by the eigenvalue > 1 and assumed 79.079% of the variance, confirming the one-dimensional model of the OES-SP as well as the scree plot.

## Discussion

There are few trials that correlate the effect of a treatment, such as dental whitening, on oral health and quality of life, psychosocial impact, and aesthetic self-perception [3]. Normally, the published papers in the literature report tooth whitening effectiveness, adverse effects, post-treatment sensitivity, and variables that result in varying concentrations of hydrogen peroxide, including adding components to reduce the sensitivity produced by whitening [28, 29]. The effect of this minimally-invasive treatment on patient-specific factors, such as perception, quality of life, and confidence, is a practically unknown area.

Furthermore, the results of the effectiveness of whitening in this study are similar to those of any previously-reported study, including the concentration and sensitivity, which are well within the frequency and intensity shown in the literature. Conventional whitening, a very popular treatment, was used as an intervention in this clinical work to assess the effect on patient-specific factors, as determined by the OHIP-Esthetics, PIDAQ, and OES questionnaires.

Evaluating the success of teeth whitening treatment has more to do with the treatment’s effectiveness in the eyes of the patient because the perception of the patient who received this treatment will ultimately be an essential determinant about whether he liked it or felt good about the change; also, this effect will not be immediate since patients often feed off of the views of close friends and associates about how they believe the whitening worked [3]. These opinions might influence the patient’s perception, trust, and psychosocial impact regarding the whitening treatment.

The whitening treatment leads to a positive impact effect in terms of the overall results of the OHIP-Esthetics questionnaire. The questionnaires were administered before and after the whitening, at one week and one month of recall, to increase the reliability of the data. The aesthetic component measured by the OHIP-Esthetics probably influenced the significant difference in the scores after one week and one month for whitening effectiveness. The positive change was evident in the self-perception of dental aesthetics at the end of the whitening and one month later, which supports the proposal that teeth whitening positively modify a patient’s self-perception of dental aesthetics. There are studies of teeth whitening that show similar OHIP results recently published in the literature that reaffirm our findings [3, 12, 30–33].

The results of the OHIP-Esthetics questionnaire at one month are quite striking because they show a significant positive effect compared to the baseline values. This could indicate that the short-term effect of whitening generates an aesthetic perception that is sharper and deeper over time than immediately after the treatment. The functional limitation dimension as such could be affected directly because the two selected questions for this shortened version of OHIP were related to the appearance of the teeth and the patient’s perception, directly affecting the function of the patient. The physical pain dimension related to tooth sensitivity or mouth could be directly related to common adverse effects of whitening treatment, with a negative impact in the case of not having any change, which would be a sign that the treatment did not generate sensitivity. The psychological discomfort dimension denotes a relationship with the welfare of or discomfort with the appearance of the teeth, and perhaps a patient seeking whitening for this factor should be one of those most marked by a positive impact.

The physical disability dimension, probably the question regarding minor flavors in foods, does not reflect the impact of whitening, and if the question of helping the patient to smile could denote a positive impact generated by lighter teeth, which may induce the patient to smile more frequently. The psychological disability dimension relates to the ability to relax or an embarrassment to show teeth; also, the second question could generate a greater impact of having lighter teeth. The social disability dimension relates to being less tolerant or having difficulties at work, which are situations that could be impacted positively as well.

The PIDAQ questionnaire was originally developed to be applied in patients receiving orthodontic treatment [11]; however, measured isolated factors also apply to a patient who experiences dental esthetics through whitening. In the first factor, “auto dental confidence,” there was a positive effect of whitening in this group of patients until the one-month recall, generating an impact of dental aesthetics on the emotional state of individuals and maintaining the effect correlated with the maintenance of color. The second factor, the social impact, also showed a positive effect immediately and at the one-month recall, referring to potential problems in social situations due to a subjective perception of an unfavorable dental appearance; the effects persisted throughout the month. The third factor of psychosocial impact is composed of items that deal with a feeling of inferiority or unhappiness when the affected individual compares him/herself to others who have superior dental aesthetics; this factor was influenced positively at all the times that were assessed. It is known that comparison processes play an important role in psychosocial well-being and that upward comparisons might provoke dysphoric moods [2]. There was a positive impact of whitening in patients maintained until the one-month recall, similar to what was reported by *Martin* et al. [3], who showed a positive impact on dental confidence via the OHIP-Esthetic questionnaire. Clearly, the PIDAQ questionnaire is a good tool to substantiate the effects of whitening. This has been poorly reported in the literature, and additional tools are needed for successful clinical treatments.

The OES was developed to assess the direct or primary aesthetic impact in prosthodontic patients and was based on patient opinion with input from dental professionals [13]. The findings support sufficient item difficulty and discrimination as well as unidimensionality in the target population. In terms of being a burden to the patient and the health care provider, the OES is easy to administer, is usually well accepted by the patient, and the scores are easy to interpret. The OES is a questionnaire that deals with questions about the perception of the face, lips, teeth, and gums and concludes with a general impression. For use in cosmetic treatments, such as whitening, this questionnaire could be useful when considering questions involving aspects of improving the smile, e.g., how to improve the perception of teeth and, in some cases, indirectly improve the perception of the soft tissues, with hardly any improvement in the questions referring to the face. In this study, there was a positive impact in addition to the overall OES score at all times regarding questions of the teeth and mouth; there was not a positive impact on the scores for questions related to the face. It would be interesting to determine whether the impact appreciated by the OES correlates with the effectiveness of whitening and long-term trials. Furthermore, the consistency and reliability of the Spanish version (OES-SP) was high, so this questionnaire could be widely used in future research.

Some limitations of this study, that arises from answering the questionnaires, i.e., the alertness of the patient or simply their interest in answering something that may not be pleasing. However, instruments such as the OHIP-Esthetics, PIDAQ and OES are widely used and have been validated by the scientific community. But there is no specific instrument to evaluate the influence of aesthetic treatments on the quality of life of people. It is also a complex aspect of studying, since there are multiple factors that influence it and are difficult to separate. These assessments have been used in many medical studies and could be beneficial tool for future researches in dentistry assess some variables forget by the trials.

## Conclusions

The at-home whitening with carbamide peroxide 10% had a positive effect on oral health quality of life, psychosocial impact and esthetics perception after one-month post-whitening. Chilean Spanish version of OES showed adequate psychometric properties to evaluate dental whitening.

## Abbreviations

Delta E: Difference or distance between two colors is a metric of interest in color science
FDI: Federation Dental International
OES: Oral Esthetics Scale
OES-SP: OES Spanish
OHIP: Oral Health Impact Profile
OHIP-14: OHIP esthetics
PIDAQ: Psychosocial Impact of Dental Esthetics Questionnaire
SGU: Shade Guide Units
WHO: World Health Organization
